# Population pharmacokinetic analysis and dosing regimen optimization of teicoplanin in critically ill patients with sepsis

**DOI:** 10.3389/fphar.2023.1132367

**Published:** 2023-04-28

**Authors:** Chao‐Yang Chen, Min Xie, Jun Gong, Ning Yu, Ran Wei, Li‐Li Lei, Si‐Miao Zhao, Ruo‐Ming Li, Xiu Dong, Xiang‐Lin Zhang, Ying Zhou, Shuang‐Ling Li, Yi‐Min Cui

**Affiliations:** ^1^ Department of Pharmacy, Peking University First Hospital, Beijing, China; ^2^ Department of Critical Care Medicine, Peking University First Hospital, Beijing, China; ^3^ Department of Pharmacy Administration and Clinical Pharmacy, School of Pharmaceutical Sciences, Peking University Health Science Center, Beijing, China; ^4^ Department of Pharmacy, China-Japan Friendship Hospital, Beijing, China; ^5^ Institute of Clinical Pharmacology, Peking University, Beijing, China

**Keywords:** teicoplanin, population pharmacokinetics, Monte Carlo simulation, dosing optimization, sepsis

## Abstract

**Objectives:** Teicoplanin has been extensively used in the treatment for infections caused by gram-positive bacteria including methicillin-resistant *Staphylococcus aureus* (MRSA). However, current teicoplanin treatment is challenging due to relatively low and variable concentrations under standard dosage regimens. This study aimed to investigate the population pharmacokinetics (PPK) characteristics of teicoplanin in adult sepsis patients and provide recommendations for optimal teicoplanin dosing regimens.

**Methods:** A total of 249 serum concentration samples from 59 septic patients were prospectively collected in the intensive care unit (ICU). Teicoplanin concentrations were detected, and patients’ clinical data were recorded. PPK analysis was performed using a non-linear, mixed-effect modeling approach. Monte Carlo simulations were performed to evaluate currently recommended dosing and other dosage regimens. The optimal dosing regimens were defined and compared by different pharmacokinetic/pharmacodynamic parameters, including trough concentration (C_min_), the ratio of 24-h area under the concentration-time curve to the minimum inhibitory concentration (AUC_0-24_/MIC), as well as the probability of target attainment (PTA) and the cumulative fraction of response (CFR) against MRSA.

**Results:** A two-compartment model adequately described the data. The final model parameter estimates for clearance, central compartment volume of distribution, intercompartmental clearance and peripheral compartment volume were 1.03 L/h, 20.1 L, 3.12 L/h and 101 L, respectively. Glomerular filtration rate (GFR) was the only covariate that significantly affected teicoplanin clearance. Model-based simulations revealed that 3 or 5 loading doses of 12/15 mg/kg every 12 h followed by a maintenance dose of 12/15 mg/kg every 24 h–72 h for patients with different renal functions were required to achieve a target C_min_ of 15 mg/L and a target AUC_0-24_/MIC of 610. For MRSA infections, PTAs and CFRs were not satisfactory for simulated regimens. Prolonging the dosing interval may be easier to achieve the target AUC_0-24_/MIC than reducing the unit dose for renal insufficient patients.

**Conclusion:** A PPK model for teicoplanin in adult septic patients was successfully developed. Model-based simulations revealed that current standard doses may result in undertherapeutic C_min_ and AUC, and a single dose of at least 12 mg/kg may be needed. AUC_0-24_/MIC should be preferred as the PK/PD indicator of teicoplanin, if AUC estimation is unavailable, in addition to routine detection of teicoplanin C_min_ on Day 4, follow-up therapeutic drug monitoring at steady-state is recommended.

## 1 Introduction

Sepsis is defined as life-threatening organ dysfunction resulting from infection (Reinhart et al., 2017), with a worldwide incidence of about 48.9 million per year in 2017 and an all-cause mortality rate with sepsis remaining 12.5%–31.8% after 2010 in developed countries (Shankar-Hari et al., 2017; Abe et al., 2018; Paoli et al., 2018; Rudd et al., 2020). Sepsis affects approximately 30% of intensive care unit (ICU) patients (Sakr et al., 2018), becoming an important issue facing critical care medicine. Methicillin-resistant *Staphylococcus aureus* (MRSA) is one of the main pathogenic bacteria in sepsis patients (David and Daum, 2010). Teicoplanin is a glycopeptide antibiotic widely used in treating infections caused by drug-resistant gram-positive bacteria, including MRSA, with comparable efficacy, improved tissue penetration, and reduced nephrotoxicity compared to other existing glycopeptides such as vancomycin (Wilson et al., 1994; Cavalcanti et al., 2010). Despite the similar mechanism of action to vancomycin, teicoplanin presents different pharmacokinetic (PK) characteristics. Unlike vancomycin, which has a plasma protein binding rate of approximately 30%–55% and a half-life of 6–12 h in adults with normal renal function, teicoplanin has a higher plasma protein binding rate (about 90%) and a longer half-life (100–170 h), enabling its administration once daily (Rowland, 1990; Rybak, 2006; Cavalcanti et al., 2010). Unchanged teicoplanin is excreted mainly by the urinary route (80% within 16 days) and only 2.7% of the dose administered is recovered in feces (*via* bile excretion) within 8 days after administration. Teicoplanin presents a low total clearance in the range of 10–14 mL/h/kg and a renal clearance in the range of 8–12 mL/h/kg, indicating that teicoplanin is mainly excreted through renal mechanisms (EMA., 2022). Currently, teicoplanin has been applied as an alternative treatment option for vancomycin, making it one of the most frequently administered antimicrobial agents in sepsis patients. However, since septic patients often present with multiple organ insufficiency and hypoalbuminemia, and are prone to PK changes such as increased volume of distribution (Ramos-Martín et al., 2017), developing an effective and safe teicoplanin dosing regimen for sepsis patients remains challenging in clinical practice. Previous studies revealed that the standard teicoplanin dosing regimens often fail to achieve adequate exposure for these patients. Additionally, optimal dosing regimens of teicoplanin for treating MRSA infections in sepsis patients have not been defined (Ueda et al., 2014; Hanai et al., 2022).

Therefore, the optimization of dosing regimens according to pharmacokinetic/pharmacodynamic (PK/PD) parameters should be recommended to improve clinical outcomes (Wei et al., 2021). As a time-dependent antibacterial drug with a long post-antibiotic effect (PAE), the PK/PD index that best correlates with teicoplanin antibacterial activity is the ratio of the 24-h area under the concentration-time curve to the minimum inhibitory concentration (AUC_0-24_/MIC) (Ramos-Martín et al., 2017). Previous studies have shown that AUC_0-24_/MIC ≥610 was a suitable PK/PD target (Ramos-Martín et al., 2017; Abdul-Aziz et al., 2020). However, because MIC detection and AUC estimation are unavailable in many institutions, teicoplanin trough concentration is recommended as a surrogate indicator in clinical practice (Harding et al., 2000; Pea et al., 2003; Gemmell et al., 2006; Kobayashi et al., 2010; Tobin et al., 2010). According to the latest consensus review by the Japanese Society of Chemotherapy and the Japanese Society of Therapeutic Drug Monitoring (TDM) (Hanai et al., 2022), a target C_min_ value of 15–30 mg/L results in better clinical efficacy and similar adverse effects in patients with non-complicated MRSA infections compared with C_min_ < 15 mg/L. Additionally, a target C_min_ value of 20–40 mg/L was recommended in patients with complicated or serious MRSA infections despite insufficient evidence. Besides the above indicators, PTA, which refers to the probability that the ratio of AUC_0-24_/MIC exceeds the target value and the cumulative fraction of response (CFR), defined as the expected population PTA for specific dosage and specific microbial population, were also applied to select the optimal dosing regimens (Zhang et al., 2020; Wei et al., 2021).

Model-informed precision dosing (MIPD) is a promising tool to guide individualized rational dosing of antibiotics based on their PK/PD targets. However, evidence supporting the MIPD approach for teicoplanin dosage calculation is relatively scarce (Hanai et al., 2022). Furthermore, though some population pharmacokinetic (PPK) studies are currently available for teicoplanin dosage optimization (Gao et al., 2020; Kontou et al., 2020; Ogami et al., 2020; Zhang et al., 2020; Aulin et al., 2021; Sako et al., 2021), scarce PPK studies have been conducted in sepsis patients (Hanada et al., 2007; Brink et al., 2015; Kontou et al., 2020). Hence, this study aimed to assess the PPK characteristics of teicoplanin and to propose an optimal teicoplanin dosage regimen in ICU patients with sepsis based on the above PK/PD indicators.

## 2 Methods

### 2.1 Study design and population

This was a prospective, open-label PPK study conducted in the ICU department of Peking University First Hospital. Patients aged ≥18 years who were diagnosed with sepsis according to Sepsis 3.0 criteria (Singer et al., 2016), confirmed or suspected with gram-positive coccal infection, and expected to be treated with teicoplanin for ≥4 days, were enrolled. Pregnant and lactating patients, patients with special sites (endocardium, bone, joints, etc.) infections, or those who received any special treatment that may affect drug elimination, such as renal replacement therapy, were excluded.

For each patient, demographic information [sex, age, height, weight, body mass index (BMI)], disease information (diagnosis, combined disease), laboratory inspection information [alanine aminotransferase (ALT), aspartate transaminase (AST), total bilirubin (TBIL), direct bilirubin (DBIL), total protein (TP), albumin (ALB), white blood count (WBC), serum creatinine (Scr), glomerular filtration rate (GFR), urea nitrogen (BUN)], medication information [dosage, formulation, administration time, dosing interval, comedication] and teicoplanin blood concentrations were accurately recorded.

### 2.2 Dosing regimen and sample collection

According to the standard dosage regimen, teicoplanin was intravenously infused at 400 mg every 12 h (q12h) for 3 doses, followed by 400 mg once daily, and subsequent adjustments were conducted according to patients’ response to the treatment. The infusion duration was 30–60 min. A 2 mL teicoplanin blood sample was collected at 4 time points (immediately before the 3rd, 4th, 5th, and 6th teicoplanin doses) from each patient. Additionally, in 1/3 patients, 2 mL more blood sample was collected immediately after the 5th infusion; in the other 1/3 patients, 2 mL more blood sample was collected 1 h after the 5th dose, and in the remaining 1/3 patients, 2 mL more concentration sample was taken 1 h before the 6th dose. All blood samples were collected in blood collection tubes anticoagulated by ethylene diamine tetra acetic acid (EDTA) for concentration determination.

### 2.3 Bioanalytical assay

Teicoplanin concentrations were detected by high-performance liquid chromatography (HPLC) with the Agilent 1,100 Series HPLC System (Agilent Technologies, Santa Clara, CA, USA). Blood samples were centrifuged within 12 h to obtain plasma. Then, 0.4 mL plasma samples were added with 0.05 mL internal standard solution (piperacillin sodium 0.15 mg/mL). Consequently, 0.6 mL acetonitrile was added, and the sample was vigorously shaken for 1 min and centrifuged at 12,000 r/min for 5 min. Then, 0.9 mL of the supernatant was transferred to a centrifuge tube, and 0.4 mL of dichloromethane was added. The solution was vigorously shaken for 1 min and centrifuged at 12,000 r/min for 5 min. The supernatant was then transferred into a vial for detection. HPLC detection conditions were as follows: Waters Symmetry C18 column (250 × 4.6 mm, 5 μm); mobile phase: 0.01 mol/L NaH_2_PO_4_ (pH = 3.3)-acetonitrile (75:25), isocratic elution; detection wavelength: 240 nm; flow rate: 1.0 mL/min; injection volume: 0.020 mL; column temperature: 35 °C (Dong et al., 2011). The limit of quantification (LOQ) of teicoplanin was 3.125 μg/mL. The above analytical method has passed the methodological verification. The concentrations of teicoplanin presented good linearity in the range of 3.125–100 μg/mL (correlation coefficient r = 0.9994). In terms of sensitivity, the signal-to-noise ratio (S/N) of the LOQ was >10. The relative standard deviations (RSDs) of the intra-day and inter-day precision of simulated plasma samples with high, medium and low concentrations were all less than 10%, and the intra-day and inter-day accuracies were 91.01%–101.13% and 92.98%–98.99%, respectively. The extraction recovery of teicoplanin was above 90%. The stability of simulated plasma samples with high, medium and low concentrations was good after being placed at room temperature for 4 h, frozen and thawed 3 times and stored at −80°C for 60 days, respectively, with the RSDs of tested samples all less than 10%.

### 2.4 PPK modelling

Teicoplanin PPK modeling was conducted using the non-linear mixed-effect modeling approach by NONMEM software (version 7.4, ICON Development Solutions).

The model was established in a stepwise manner. In the basic model screening process, one-, two- and three-compartment models with additive, proportional and mixed error were tested respectively, and the basic model was selected by the objective function value (OFV) and visual diagnostic plots (Gao et al., 2020). The model parameters were assumed to follow a log-normal distribution, and the inter-individual variability for each structural parameter was modeled using the following equation:

$$P_{i}=P_{TV}\times \mathrm{Exp}\;\left(\eta_{i}\right)$$ (1)

where *P*_*i*_ represents parameters for the *i*_*th*_ individual, *P*_*TV*_ are the typical values of the parameters, and *η*_*i*_ are random variables with zero mean and variance of ω^2^.

Demographic and clinical characteristics that were considered plausible for affecting teicoplanin PK were tested as covariates, including sex, age, height, weight, BMI, ALT, AST, TBIL, DBIL, TP, ALB, WBC, Scr, GFR, and BUN. Individual PK estimates obtained from the selected structural model were firstly plotted against covariate values to assess relationships (Byrne et al., 2017). When a relationship between the covariate and the PK parameter was observed, the covariate was tested for inclusion into the PPK model. Continuous covariates were incorporated into the model by the following formula (Cazaubon et al., 2017):

$${CL}_{i}={CL}_{pop}\times {\left({COV}_{i}/{COV}_{median}\right)}^{\beta }\times e^{\eta_{CL,i}}$$ (2)

where CL_*i*_ represents the CL of the i_th_ subject, CL_pop_ is the population parameter estimate, COV_*i*_ is the covariate value of subject i, COV_median_ is the median value of the covariate in the study population, β is the covariate effect to be estimated, and η_CL,*i*_ represents the individual random effect.

For binary covariates, the formula was:

$${CL}_{i}={CL}_{pop}\times e^{\beta \cdot COVi}\times e^{\eta_{CL,i}}$$ (3)

where the value of COV_*i*_ is 0 or 1.

If the inclusion of the covariates resulted in a decrease of >3.84 in the OFV (*P* = 0.05, χ^2^ distribution with one degree of freedom), it was supported for inclusion in the full regression model (Roberts et al., 2015). After that, the covariates were removed one by one from the full regression model, and covariates resulting in an increase of >10.828 in the OFV (*P* = 0.001, χ^2^ distribution with one degree of freedom) were retained in the final model.

### 2.5 Model diagnostics

The performance of the final model was evaluated by internal validations, including goodness-of-fit (GOF), bootstrap and prediction corrected visual predictive check (pc-VPC). GOF evaluation was performed by plotting the corresponding individual (IPRED) and population predictive values (PRED) against the observed values as well as the PRED and time against conditional weighted residual errors (CWRES). A bootstrap resampling technique was used for model validation. One thousand bootstrap-resampled data sets were generated from the original model group data set, and each was individually fitted to the final model. All parameters were estimated, and the median and 95% confidence intervals (CIs) (2.5th percentile and 97.5th percentile) were compared with the final parameter estimates. Pc-VPC was used to graphically assess the appropriateness of the compartment model based on 1,000 replicates of the dataset (Bergstrand et al., 2011).

### 2.6 Dosage simulations

To provide recommendations for teicoplanin dosage selection, Monte Carlo simulations were performed using the final PPK model with GFR covariate to generate PK profiles for candidate dosage regimens. Trough concentrations on Day 4 (C_min, 72h_) and at steady state 7 days after the initial TDM (Day 11, C_min, 240h_) (Hanai et al., 2022) and the AUC_0-24_ were obtained from each condition. IV teicoplanin loading doses ranging from 10–15 mg/kg, administered every 12 h for either 3 or 5 doses with maintenance doses ranging from 3–15 mg/kg, administered per 24–72 h to a typical 65 kg patient were studied. Five GFR levels of 15, 30, 60, 90 and 120 mL/min/1.73 m^2^ were simulated. At each GFR level, 1,000 simulations were performed for each candidate regimen. The concentration-time profile was simulated between 0 and 264 h.

AUC_0-24_ values estimated by the linear trapezoidal method from the concentration-time profiles were divided by putative MIC values to obtain AUC_0-24_/MIC ratios. According to the European Committee on Antimicrobial Susceptibility Testing (EUCAST, https://mic.eucast.org/Eucast2/) data, MIC values of 0.25, 0.5, 1, 2, and 4 were selected. PTAs for achieving an AUC_0-24_/MIC of ≥610 (Ramos-Martín et al., 2017; Abdul-Aziz et al., 2020) were calculated. Based on the MIC distributions for MRSA reported by the EUCAST, CFR was calculated with the following formula (Wei et al., 2021) to define the optimal dosage regimens attaining the AUC_0-24_/MIC target of 610:

$$CFR=\sum_{i=1}^{n}PTA\left(MICi\right)\cdot p\left(MICi\right)$$ (4)

where PTA(MIC*i*) is the PTA value at the i_th_ MIC category of MRSA, and *p* (MIC*i*) is the fraction of isolates of MRSA at this MIC category.

A dosing regimen resulting in an AUC_0-24_/MIC value of ≥610, a PTA value of ≥90%, a C_min_ value of 15–30 mg/L in non-complicated MRSA infectious patients was considered to be the optimal regimen. The regimen that achieved ≥90% CFR was considered the optimal empirical therapy, while a CFR between 80% and 90% was associated with a moderate probability of success (Bradley et al., 2003).

## 3 Results

### 3.1 Subject characteristics

In this prospective study, 249 plasma samples were collected from 59 subjects (22 females and 37 males). The median (range) age and weight were 72.0 (28.0–92.0) years and 65.0 (35.0–90.0) kg, respectively. Among the included patients, 23.7% had normal kidney function (GFR ≥90 mL/min/1.73 m^2^), 33.9% had mildly impaired renal function (GFR between 60 and 90 mL/min/1.73 m^2^), 23.7% had moderately impaired renal function (GFR between 30 and 60 mL/min/1.73 m^2^), 8.5% had severely impaired renal function (GFR between 15 and 30 mL/min/1.73 m^2^), and 10.2% of the patients had kidney failure (GFR <15 mL/min/1.73 m^2^). A summary of the characteristics of the included patients is provided in Table 1.

**TABLE 1:** Characteristics of the patients.

Variable	Value (N = 59)
Number of enrolled patients	59
Gender (male, number (%))	37 (62.7%)
Age (years) (median (range))	72.0 (28.0–92.0)
Height (m) (median (range))	1.67 (1.33–1.80)
Weight (kg) (median (range))	65.0 (35.0–90.0)
BMI (kg/m^2^) (median (range))	24.1 (15.6–32.0)
ALT (IU/L) (median (range))	15.0 (4.00–993)
AST (IU/L) (median (range))	26.0 (11.0–2,390)
TBIL (μmol/L) (median (range))	18.7 (5.50–244)
DBIL (μmol/L) (median (range))	6.80 (0.600–141)
TP (g/L) (median (range))	52.3 (33.5–71.0)
ALB (g/L) (median (range))	29.5 (15.5–39.4)
WBC (×10^9^/L) (median (range))	11.5 (2.39–25.9)
Scr (μmol/L) (median (range))	80.3 (28.4–429)
GFR (mL/min/1.73m^2^) (median (range))	71.9 (11.0–124)
BUN (mmol/L) (median (range))	10.5 (1.09–81.6)

BMI were calculated by the following formula: BMI (kg/m^2^) = weight (kg)/[height (m)]^2^.

### 3.2 PPK model building

During the modeling process, one-, two- and three-compartment models with additive, proportional and mixed residual variability were evaluated to describe teicoplanin’s PK characteristics, and a two-compartment model with proportional residual best described the data. The first-order conditional estimation method with the η-ε interaction option was adopted, and the ADVAN3, TRANS4 subroutines were selected in NONMEM software. The proportional residual variability was expressed as Equation 5.

$$C_{ij}=C_{pred,ij}\times \left(1+\varepsilon_{prop,ij}\right)$$ (5)

where *C*_*ij*_ is the *j*_*th*_ observation for the *i*_*th*_ subject, *C*_*pred,ij*_ is the *j*_*th*_ predicted value for the *i*_*th*_ subject, and *ε*_*prop,ij*_ is the proportional residual of the measured concentrations, with means of 0 and variances of σ_prop_^2^.

Covariate relationship plots and the impact of covariates on the Bayesian posthoc PK parameters from the final PPK basic model are provided in Supplementary Figure S1 and Supplementary Table S1 (available as Supplementary Data). After covariate screening, GFR was found to significantly influence teicoplanin CL, resulting in a decrease of 17.95 in OFV and a reduction of 9.8% in ω_CL_, and was the only covariate included in the final model. The final model parameter estimates for CL, V1, Q, and V2 were 1.03 L/h, 20.1 L, 3.12 L/h, and 101 L, respectively (Table 2). The final model equation was as follows:

$$CL\;\left(L/h\right)=1.03\times {\left(\frac{GFR}{71.88}\right)}^{0.437}\times e^{0.29}$$ (6)

$$V1\;\left(L\right)=20.1\times e^{0.37}$$ (7)

$$Q\;\left(L/h\right)=3.12\times e^{0.29}$$ (8)

$$V2\;\left(L\right)=101\times e^{0.10}$$ (9)

**TABLE 2:** Parameter estimates, standard error, and bootstrap confidence intervals of the final model.

	Estimate (RSE%)	923 successful bootstrap median (95% PI)
PK Parameter		
CL, (L/h)	1.03 (16.6)	1.05 (0.818–1.301)
V1, (L)	20.1 (12.9)	19.8 (16.1–26.2)
Q, (L/h)	3.12 (10.9)	3.14 (2.62–3.90)
V2, (L)	101 (12.7)	98.2 (77.5–122.2)
$\theta_{CL\_GFR}$	0.437 (23.8)	0.441 (0.234–0.740)
Interindividual variability		
CL, %	53.9 (10.1)	51.5 (37.1–63.3)
V1, %	60.7 (23.9)	58.9 (36.2–91.8)
Q, %	54.3 (15.5)	54.2 (38.9–73.0)
V2, %	32.1 (16.0)	32.2 (18.7–47.5)
Residual error		
Proportional error, %	17.4 (0.00286)	16.5 (11.8–20.9)

### 3.3 Model validation

**FIGURE 1:**
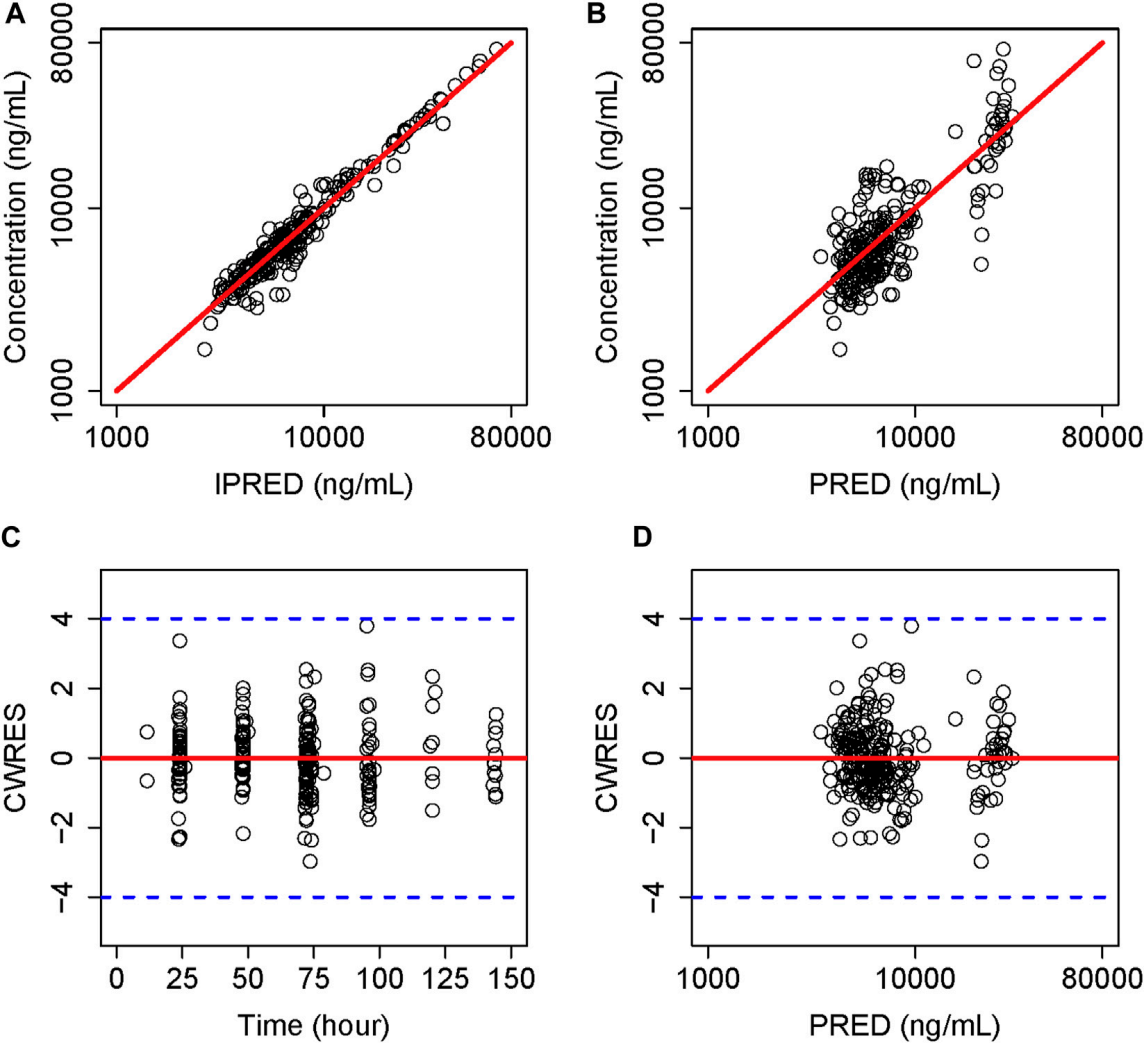
Goodness-of-fit plot of the final model. **(A)** Individual predicted concentration *versus* observed concentration. **(B)** Population predicted concentration *versus* observed concentration. **(C)** Conditional weighted residuals *versus* time. **(D)** Conditional weighted residuals *versus* population predicted concentration. The red lines in **(A,B)** represent the regression line, while the solid red lines in **(C,D)** indicate the position where conditional weighted residual equals 0.

**FIGURE 2:**
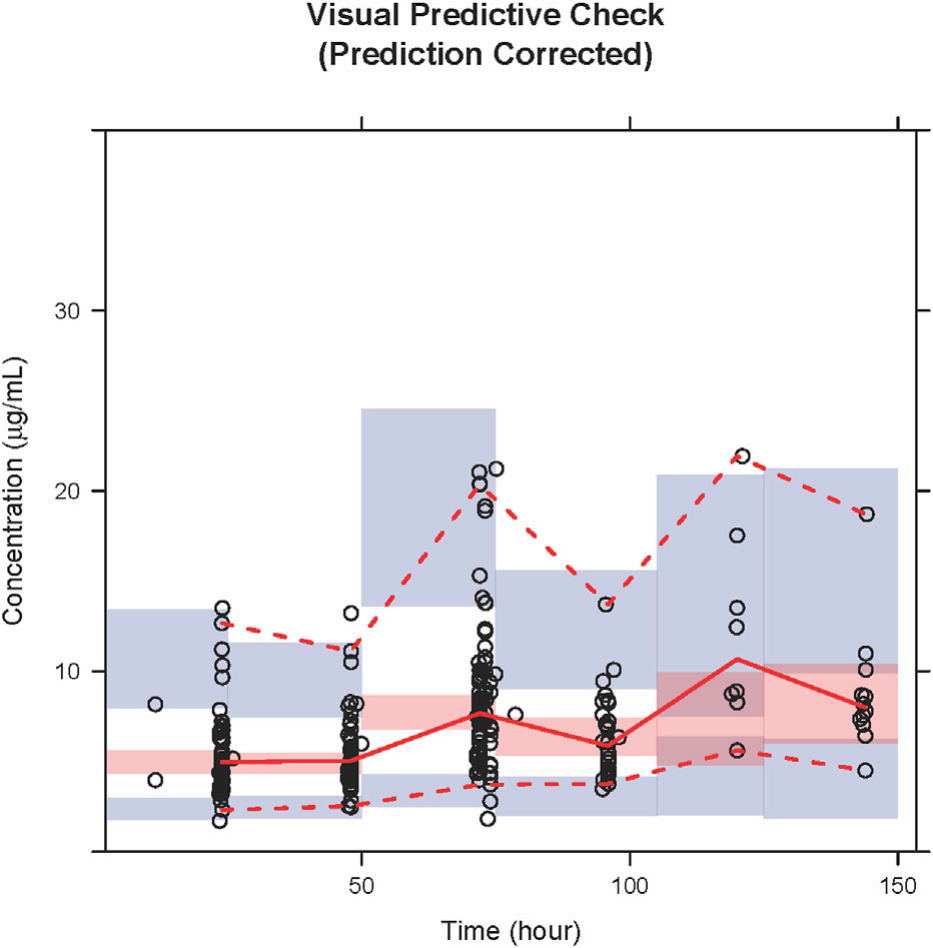
PcVPC results of the final model. Open circles represent the observed concentrations, and the solid line and dashed lines represent the observations’ median and 95% CI. The middle red shadow areas represent the 95% CI of medians, and the blue shadow areas represent the 95% CI of the 2.5th and 97.5th percentiles of the results of a 1,000 simulation in the final model.

The GOF plots for the final model illustrated that PRED and IPRED were in good accordance with the observed concentrations (Figure 1). The CWRES showed good scattering, with all points between ±4. The final model demonstrated strong stability, with 923 out of 1,000 bootstrap runs fitting successfully, and the parameter estimates were similar to the bootstrap median estimates meanwhile were within the 95% percentile interval (PI) of the bootstrap simulation results (Table 2). The pc-VPC results confirmed a sufficient predictive power of the final teicoplanin model, with the observed data included in the range of 95% CI and the median and 95% CI lines of the observations located near the middle of the 1,000 simulation results (Figure 2).

### 3.4 Monte Carlo simulation

Table 3 demonstrates the median and variability of the C_min_ and AUC_0-24_/MIC simulation results achieved with the various dosage regimens. Only the AUC_0-24_/MIC and recommended regimens when MIC = 1 are presented in Table 3 since the results are applicable to most cases with MIC ≤1, and AUC_0-24_/MIC at other MIC levels can be calculated accordingly. As expected, teicoplanin C_min_ and exposure generally increased with increasing dosage and decreasing renal function. As seen in the table, the results of loading dosage regimens were mainly reflected in C_min, 72h_, while the results of the maintenance dosage regimens were mainly reflected in C_min, 240h_ and AUC_0-24_/MIC. Taking the simulated patients with a GFR of 120 mL/min/1.73 m^2^ as an example, though the simulated C_min, 72h_ under the second dosage regimen was higher than that of the first due to the higher frequency of the loading doses (15 mg/kg q12h*5 vs. 15 mg/kg q12h*3), the C_min, 240h_ and AUC_0-24_/MIC were less than that due to the lower maintenance doses [7.5 vs. 15 mg/kg every 24 h (q24h)]. Besides, for patients with renal insufficiency (simulated GFR of 60, 30, 15 mL/min/1.73 m^2^), extending the dosing interval seemed more likely to achieve target PK/PD results than reducing the unit dosage. Based on the above simulations, for patients with a GFR of 90–120 mL/min/1.73 m^2^, the recommended dosing regimen was 15 mg/kg q12h for 5 times, followed by 15 mg/kg q24h. For patients with a GFR of 30–60 mL/min/1.73 m^2^, a dosage regimen of 15 mg/kg q12h for 3 times followed by 15 mg/kg on Day 3 and 15 mg/kg q48h from Day 4 was able to make the teicoplanin C_min_ and exposure achieve the targets. For patients with a GFR of 15 mL/min/1.73 m^2^, a regimen of 12 mg/kg q12h for 3 times followed by 12 mg/kg on Day 3 and 12 mg/kg q72h from Day 4 was enough to make the above parameters meet the standard (Table 3).

**TABLE 3:** Monte Carlo simulation results stratified by GFR.

GFR (mL/min)	Dosing regimen				C_min,72h_ (mg/L) Median [95% PI]	C_min,240h_ (mg/L) Median [95% PI]	AUC_0-24_/MIC^a^ Median [95% PI]
	Day1	Day2	Day3	Day4			
120	15 mg/kg q12h	15 mg/kg q24h	15 mg/kg q24h	15 mg/kg q24h	12.18 [6.383–22.62]	19.04 [7.152–39.28]	687 [286.3–1,436]
	15 mg/kg q12h	15 mg/kg q12h	15 mg/kg q24h	7.5 mg/kg q24h	15.45 [7.833–27.65]	12.12 [3.865–27.24]	413.9 [149–927.5]
	**15 mg/kg** ^b^ **q12h**	**15 mg/kg q12h**	**15 mg/kg q24h**	**15 mg/kg q24h**	**15.11 [7.911–27]**	**19.87 [6.637–41.51]**	**703 [264.4–1,550]**
90	15 mg/kg q12h	15 mg/kg q24h	15 mg/kg q24h	15 mg/kg q24h	13.09 [6.768–23.5]	21.28 [7.764–41.54]	734.7 [272.9–1,514]
	15 mg/kg q12h	15 mg/kg q12h	15 mg/kg q24h	7.5 mg/kg q24h	16.73 [9.037–30.6]	13.57 [4.469–30.34]	446.9 [160.7–1,005]
	**15 mg/kg q12h**	**15 mg/kg q12h**	**15 mg/kg q24h**	**15 mg/kg q24h**	**16.91 [9.048–30]**	**22.18 [8.989–46.77]**	**775.7 [316.1–1,651]**
60	15 mg/kg q12h	15 mg/kg q24h	15 mg/kg q24h	7.5 mg/kg q24h	15.15 [7.984–27.29]	15.37 [5.58–31.3]	497.7 [191.3–1,007]
	**15 mg/kg q12h**	**15 mg/kg q24h**	**15 mg/kg q24h**	**15 mg/kg q48h**	**14.74 [8.032–26.02]**	**16.93 [6.484–33.92]**	**625.2 [240.5–1,348]**
**30**	**15 mg/kg q12h**	**15 mg/kg q24h**	**15 mg/kg q24h**	**7.5 mg/kg q24h**	**17.94 [9.903–32.1]**	**20.13 [7.541–39.91]**	**618.2 [239.1–1,254]**
	**15 mg/kg q12h**	**15 mg/kg q24h**	**15 mg/kg q24h**	**15 mg/kg q48h**	**17.97 [9.926–31.05]**	**22.33 [8.65–42.9]**	**761.2 [295.6–1,646]**
**15**	**12 mg/kg** ^c^ **q12h**	**12 mg/kg q24h**	**12 mg/kg q24h**	**12 mg/kg q72h**	**16.28 [8.994–28.07]**	**19.08 [7.829–36.81]**	**647.7 [242.1–1,312]**
	15 mg/kg q12h	15 mg/kg q24h	15 mg/kg q24h	5 mg/kg q24h	20.27 [11.64–35.46]	20.66 [8.496–38.14]	587.6 [250.2–1,118]
	**15 mg/kg q12h**	**15 mg/kg q24h**	**15 mg/kg q24h**	**15 mg/kg q72h**	**20.26 [11.66–36.69]**	**23.72 [10.97–45.7]**	**802.3 [342.5–1,686]**

^a^ MIC = 1 mg/L.

^b^ 15 mg/kg equates to approximately 1,000 mg for the convenience of clinical operation.

^c^ 12 mg/kg equates to approximately 800 mg for the convenience of clinical operation; The bold fonts indicate dosage regimens that both meet the C_min, 72h_, C_min, 240h_ and AUC_0-24_/MIC standard.

**FIGURE 3:**
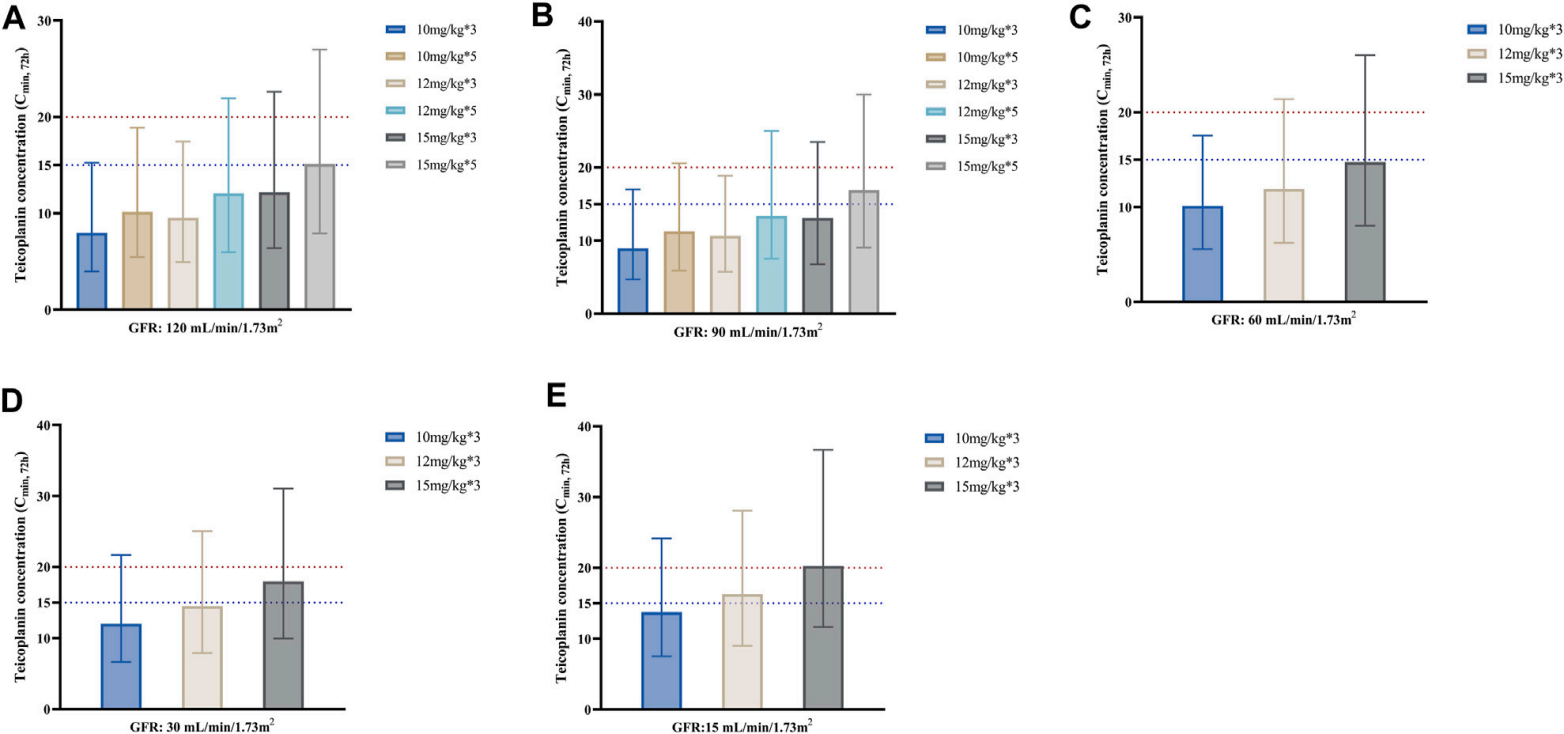
Median teicoplanin C_min, 72h_ with different loading doses in subgroups stratified by GFR. **(A–E)** represent subgroups with GFR of 120, 90, 60, 30 and 15 mL/min/1.73m^2^, respectively. Each bar represents the median with a 95% percentile interval (PI). Loading doses were administered every 12 h, and C_min_ was simulated by day 4 (72 h). The dashed blue and red lines indicate the target C_min_ of 15 mg/L (non-complicated infections) and 20 mg/L (complicated infections), respectively.

Figure 3 shows the median C_min, 72h_ achieved with different loading dose regimens in subgroups stratified by GFR. As seen from the figure, for the same unit dose, increasing the number of loading doses may significantly elevate teicoplanin trough concentrations at 72 h for a faster attainment of the target (Figures 3A, B). Generally, lower loading doses were required in patients with lower GFR. For patients with GFR 90–120 mL/min/1.73 m^2^, a loading dose of 15 mg/kg for 5 times made it possible to achieve a C_min, 72h_ of ≥15 mg/L; for patients with GFR 30–60 mL/min/1.73 m^2^, a loading dosage regimen of 15 mg/kg for 3 times was enough, while for patients with GFR 15 mL/min/1.73 m^2^, the loading dosage may be reduced to 12 mg/kg for 3 times in patients with non-complicated infections.

**FIGURE 4:**
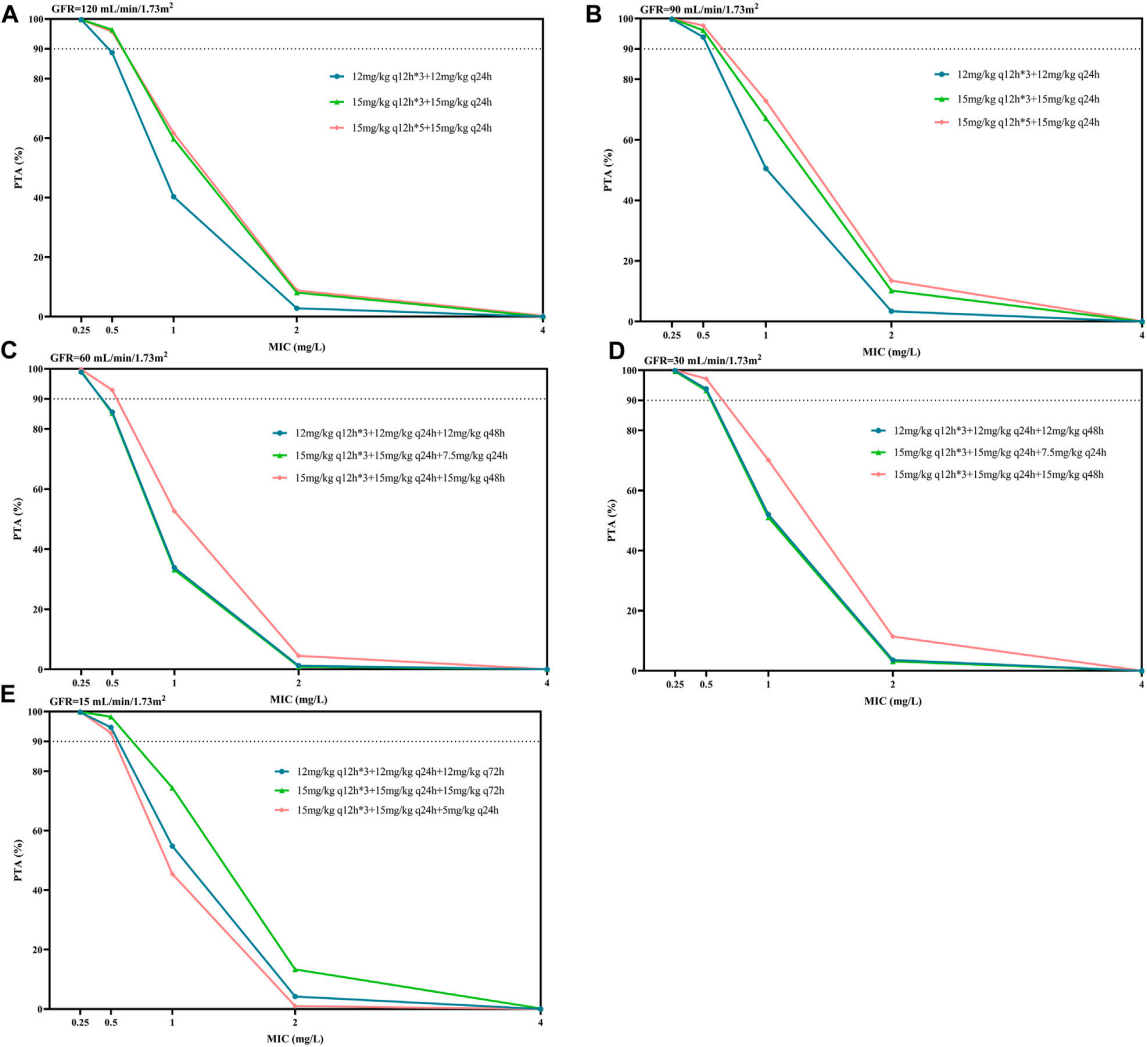
PTA of teicoplanin against MRSA for the target of AUC_0‐24_/MIC ≥ 610 in subgroups stratified by GFR. **(A–E)** represent subgroups with GFR of 120, 90, 60, 30 and 15 mL/min/1.73m^2^, respectively. The dashed black line indicates the target PTA of 90%.

Figure 4 displays the PTA against MRSA associated with the PK/PD target of efficacy, AUC_0-24_/MIC ≥610. According to the EUCAST data, most MRSA had an MIC distribution for teicoplanin of 0.5–1 mg/L. When considering a target of PTA ≥90%, we found that the conventional dosing regimen of 400 mg (6 mg/kg) or 600 mg (10 mg/kg) q24h was not adequate for the simulated patients (not listed in Figure 4). For patients with GFR 90–120 mL/min/1.73 m^2^, two regimens with a maintenance dose of 15 mg/kg q24h resulted in similar PTAs, which were higher than the maintenance dose of 12 mg/kg q24h. Consistent with the trend in Table 3, for patients with GFR 30–60 mL/min/1.73 m^2^, the PTAs of the maintenance dose 15 mg/kg q48h regimen was higher than that of the maintenance dose 7.5 mg/kg q24h; the latter was similar to the maintenance dose of 12 mg/kg q48h. For patients with GFR 15 mL/min/1.73 m^2^, the PTA values obtained by the three maintenance schemes of 15 mg/kg q72h, 12 mg/kg q72h, and 5 mg/kg q24h gradually decreased, which also confirmed that extending the dosing interval might be easier to achieve the PTA target than reducing the unit dose for patients with renal insufficiency. Generally, for patients with lower GFR, lower maintenance doses were required to achieve PTA targets. For MRSA isolates, the vast majority of the listed regimens achieved PTA targets when MIC = 0.5 mg/L; however, no dosage achieved 90% of PTA when MIC = 1 mg/L (Figure 4).

**FIGURE 5:**
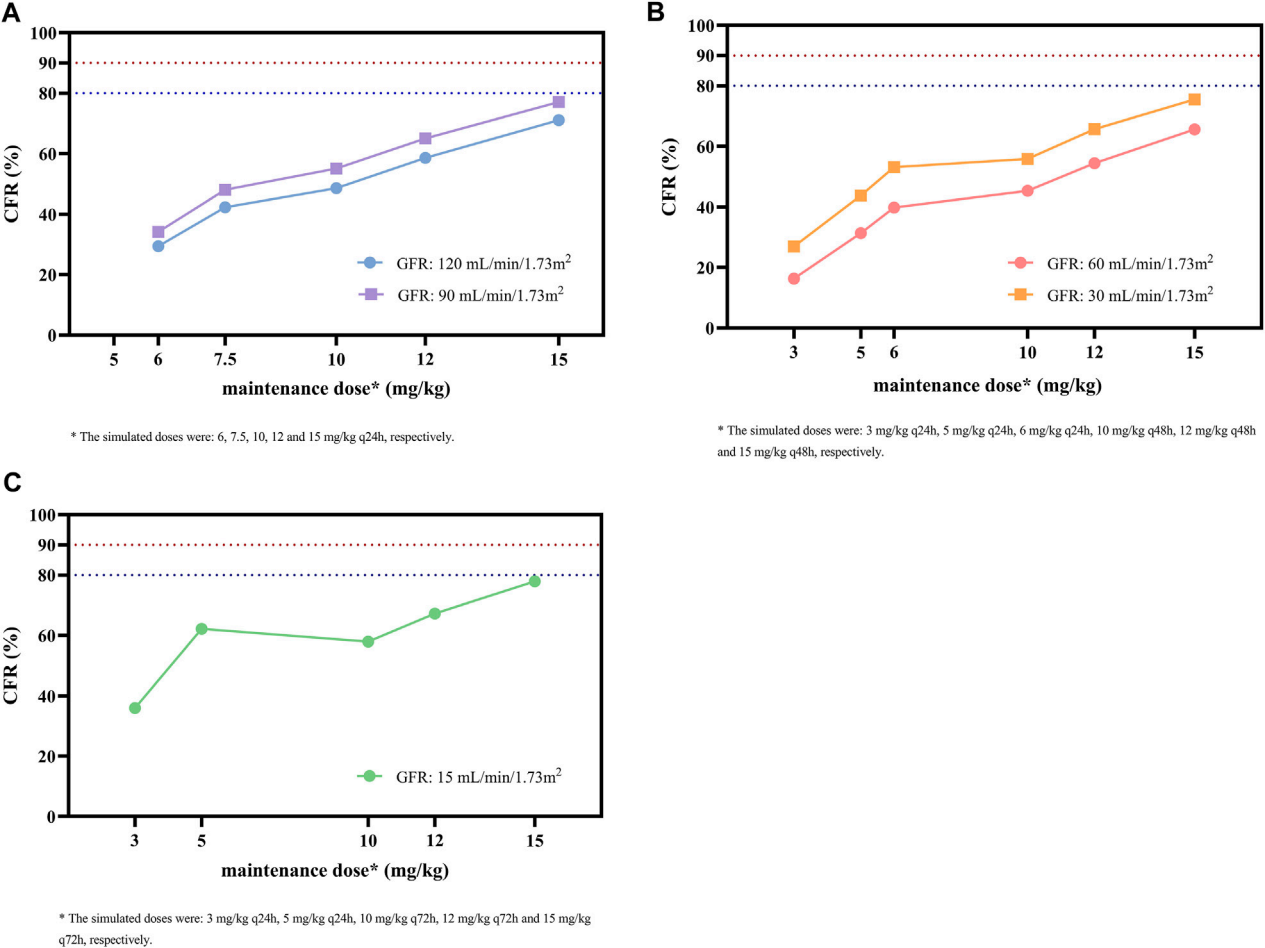
CFR of different maintenance doses against MRSA for the target of AUC_0-24_/MIC ≥ 610 in subgroups stratified by GFR. **(A)** Subgroups with GFR of 120 and 90 mL/min/1.73 m^2^. **(B)** Subgroups with GFR of 60 and 30 mL/min/1.73m^2^. **(C)** Subgroup with GFR of 15 mL/min/1.73 m^2^. The MIC range and distribution are based on the EUCAST data published in 2022. The dashed blue and red lines indicate the target CFR of 80% and 90%, respectively.

As shown in Figure 5, the simulated dosing regimens of 15 mg/kg q24h in patients with a GFR of 90 mL/min/1.73 m^2^, 15 mg/kg q48h in patients with a GFR of 30 mL/min/1.73 m^2^, and 15 mg/kg q72h in patients with a GFR of 15 mL/min/1.73 m^2^ almost achieved CFR values of ≥80% for the target of AUC_0-24_/MIC ≥610. However, none of the simulated dosing regimens could make CFR values exceed 90%. For the same simulated regimen, the lower GFR level of patients might result in higher CFR values.

## 4 Discussion

To our knowledge, this is the first PPK model of teicoplanin in ICU adult patients with sepsis established by prospective blood collection data covering the absorption, distribution, metabolism, and excretion phases. Monte Carlo simulation based on PK/PD theory rationalized and optimized dosing regimens for teicoplanin in septic patients with different renal function levels, which partially filled the gap in teicoplanin instructions, as the instruction does not have a dosage recommendation for patients with sepsis (EMA., 2022).

The final model was a two-compartment model with GFR as the only covariate significantly affecting teicoplanin CL. The final model parameter estimates for CL, V1, Q, and V2 were 1.03 L/h, 20.1 L, 3.12 L/h, and 101 L, respectively. The present model was similar to the previously published model by Wi et al. (Wi et al., 2017) in the typical value of CL (1.03 vs. 0.95 L/h), although the distribution was larger (20.1 L vs. 15.7 L in V1; 101 L vs. 71.7 L in V2). This difference was expected, since factors such as endothelial damage, increased capillary permeability, compounded by a large amount of fluid collections in various body cavities in septic patients might increase the distribution volume (Fujii et al., 2020; de Cacqueray et al., 2022). Covariate analysis in this study demonstrated that GFR calculated by the CKD-EPI equation (Levey et al., 2009) was the only significant covariate affecting teicoplanin PK, which is similar to the previous study (Cazaubon et al., 2017) and consistent with the fact that teicoplanin is mainly eliminated unchanged by the kidney (Falcoz et al., 1987). Several studies revealed creatinine clearance and weight as significant covariates influencing teicoplanin elimination (Byrne et al., 2017; Byrne et al., 2018; Kontou et al., 2020); however, weight in this study population did not seem to have a significant effect on teicoplanin, which was consistent with previous PPK studies from Lortholary *et al.* (Lortholary et al., 1996) and Cazaubon *et al.* (Cazaubon et al., 2017). A good stability and accuracy of the final model were confirmed by GOF, bootstrap, and pc-VPC estimates.

Considering the high plasma protein binding rate (about 90%) and extremely long elimination half-life of teicoplanin, a loading regimen is recommended to achieve target C_min_ rapidly during treatment (Pea et al., 2003; Yoon et al., 2014; Wang et al., 2015), which is crucial in the treatment of severe infections, especially in critically ill patients (Kollef, 2001; Niederman, 2003; Kollef, 2013). According to teicoplanin instruction (EMA., 2022), the recommended loading dosage depends on the indication: 12 mg/kg q12h for 3 to 5 administrations for bone and joint infections and endocarditis, while 6 mg/kg q12h for 3 administrations for other infections. However, a study by Cazaubon *et al.* (Cazaubon et al., 2017) revealed that a loading dose regimen with 5 administrations of either 400 (approximately 6 mg/kg) or 600 mg (approximately 9 mg/kg) was not sufficient to achieve the target C_min, 72h_ ≥ 15 mg/L in most patients, and at least 800 mg (approximately 12 mg/kg) should be used to achieve this target with a PTA ≥90%. This finding was in agreement with previous research (Brink et al., 2008; Kato et al., 2016; Nakano et al., 2016). Similar conclusions were obtained in this study in septic patients, suggesting that at least 800 mg (about 12 mg/kg) is required to achieve the target of C_min, 72h_ ≥ 15 mg/L regardless of patients’ renal function (Figure 3). Besides, the present study revealed that increasing the dosing frequency was even more likely to enhance teicoplanin trough concentrations at 72 h than increasing unit doses (take the C_min, 72h_ of 10 mg/kg for 5 administrations compared to 12 mg/kg for 3 administrations in Figure 3A as an example).

Higher loading doses can provide higher drug exposure at the start of treatment, but the difference appears to diminish after 14 days when different loading doses are followed by the same maintenance dose, therefore, a sufficient maintenance dose seems important (Ahn et al., 2011). Maintenance dose is mainly reflected in C_min, 240h_ and AUC_0-24_/MIC, according to our simulation, at low maintenance doses such as 5 mg/kg or 7.5 mg/kg, neither C_min, 240h_ nor AUC_0-24_/MIC could reach the target (GFR 90 and 120 mL/min/1.73 m^2^, maintenance dose 7.5 mg/kg q24h, Table 3); even when C_min, 240h_ exceeded the target value, AUC_0-24_/MIC still could not meet the standard (GFR 60 mL/min/1.73 m^2^, maintenance dose 7.5 mg/kg q24h; GFR 15 mL/min/1.73 m^2^, maintenance dose 5 mg/kg q24h, Table 3). Additionally, our data found that for renal insufficient patients, extending the dosing interval was more likely to bring the AUC_0-24_/MIC to the target than reducing the unit dose, though the concentrations obtained by both methods may be similar (GFR 15, 30, and 60 mL/min/1.73 m^2^, Table 3). The reason may be that the PK/PD index for teicoplanin was AUC for 0–24 h, while extending the dosing interval mainly affects teicoplanin exposure during the elimination phase, hence the effect on AUC_0-24_ is not as intuitive as the effect of reducing the unit dose. This provides some new information, as in teicoplanin instruction (EMA., 2022), both decreasing unit dose or prolonging dosage interval are recommended for renal insufficient patients, but whether the two methods may cause a difference in teicoplanin 0–24 h exposure is not mentioned. Our finding is consistent with the review by Gilbert (Gilbert et al., 2009), in which extending the dosing interval was the only recommendation for teicoplanin administration in patients with renal failure. The findings also suggested that for teicoplanin, AUC_0-24_/MIC may be a more sensitive PK/PD indicator than trough concentration.

Though C_min_ is a common index for glycopeptide TDM and dose optimization, it does not take bacterial susceptibility into account, which is considered to affect the clinical outcome (Lodise et al., 2008). Therefore, in this study, we also calculated the AUC_0-24_/MIC, PTA, and CFR as targets based on evidence suggesting that these indicators were associated with both bacteriological efficacy and prevention of drug resistance (Rose et al., 2008; Matsumoto et al., 2016). Since C_min, 72h_, which is recommended as a surrogate measure for teicoplanin TDM, is correlated with loading dosage, whereas AUC_0-24_/MIC is more closely related to maintenance dosage, the optimal dosing regimens obtained by the above two indicators were slightly inconsistent, but the overall difference was not significant (Table 3). The simulation results showed that the PTA was above 90% for most of the simulated dosages against MRSA with an MIC of 0.5 mg/L; however, it decreased to 33.1%–74.4% at an MIC of 1 mg/L in patients with varying renal function with the index of AUC_0-24_/MIC ≥610, and no simulated regimen was able to get the PTA to the 90% target (Figure 4). In addition, CFR results showed the teicoplanin unit dose of 15 mg/kg q24h in patients with GFR 90 mL/min/1.73 m^2^, 15 mg/kg q48h in patients with GFR 30 mL/min/1.73 m^2^, and 15 mg/kg q72h in patients with GFR 15 mL/min/1.73 m^2^ allowed patients to achieve an acceptable CFR of nearly 80%; similarly, none of the simulated regimens could bring the CFR to 90% target (Figure 5).

Compared to C_min_, AUC-guided dosing is preferable if conditions permit (Hanai et al., 2022), since AUC_0-24_/MIC is the recommended PK/PD index for such time-dependent antibiotics with long PAEs and responds to the overall exposure of teicoplanin. However, due to obstacles such as lack of practitioner familiarity, time allocation and training requirements, AUC-based dosing is unavailable in many clinical settings, in this case, C_min_ may be recommended as a surrogate marker (Zhang et al., 2020). Therefore, the two indexes of C_min_ and AUC_0-24_/MIC were both included in this study, and the results of each index were reported, for the selection and reference of clinical practitioners according to their own conditions. Though C_min,72h_ on Day 4 before reaching steady state is recommended as a surrogate measure for teicoplanin TDM, the value is mainly an evaluation of the loading dose for the initial 3 days, therefore follow-up TDM such as C_min, 240h_ is suggested to be conducted to evaluate the maintenance dose (Hanai et al., 2022). Taken together, optimal dosage regimens based on both C_min_ and AUC_0-24_/MIC targets in this study are recommended in order to achieve both microorganism-nonspecific and microorganism-specific targets, which are 5 loading doses of 15 mg/kg (equates to approximately 1,000 mg) q12h followed by a maintenance dose of 15 mg/kg q24h for patients with a GFR of 90–120 mL/min/1.73 m^2^; 3 loading doses of 15 mg/kg (equates to approximately 1,000 mg) q12h followed by a maintenance dose of 15 mg/kg on Day 3 and 15 mg/kg q48h from Day 4 for patients with a GFR of 30–60 mL/min/1.73 m^2^, and 3 loading doses of 12 mg/kg (equates to approximately 800 mg) q12h followed by a maintenance dose of 12 mg/kg on Day 3 and 12 mg/kg q72h from Day 4 for patients with a GFR of 15 mL/min/1.73 m^2^ (Table 3). In addition, since the PTA and CFR results obtained from the simulated regimen in this study were not very satisfactory, the clinical efficacy of the recommended regimen should be verified by further real-world studies.

To our knowledge, this is the first PPK model developed for teicoplanin in ICU adult patients with sepsis. Dosing regimens were optimized by four targets, including C_min_, AUC_0-24_/MIC, PTA and CFR, making it possible to compare the results directly from the above targets widely adopted in dose optimization and to understand the differences between them. The priority of the indicators and the limitation of C_min,72h_ as a surrogate metric were also discussed. Monte Carlo simulations provided new information to the dose optimization strategy of patients with renal failure, indicating that AUC_0-24_/MIC targets are more likely to be achieved by extending the dosing interval than by reducing the unit dose. Finally, based on the above simulation results, this study proposed a dose selection scheme of teicoplanin for septic patients with different renal functions taking into account both C_min_ and AUC_0-24_/MIC targets.

This study has several limitations. First, the analysis was based on data obtained from ICU septic patients, therefore, the conclusions may not necessarily apply to other patient populations. Besides, we adopted putative MIC values in the simulations since individual MIC values were not available. Finally, considering that the PTA and CFR results of the simulated dose schemes were not perfect, clinical studies are necessary to assess the safety and clinical utility of teicoplanin doses higher than currently recommended.

## 5 Conclusion

In conclusion, we successfully developed a PPK model for teicoplanin based on a prospective cohort of Chinese septic patients. Model-based simulations suggest that the current standard protocol may result in undertherapeutic C_min_ and AUC, and a single dose of at least 12 mg/kg is required. Regarding patients with renal insufficiency, prolonging the dosing interval rather than reducing the unit dose is recommended to achieve the target AUC_0-24_/MIC. Though trough concentration on Day 4 is the widely used TDM indicator for teicoplanin, it is recommended that follow-up steady-state trough concentration should be considered to reflect the maintenance dose of teicoplanin treatment.

## Data Availability

The original contributions presented in the study are included in the article/Supplementary Material, further inquiries can be directed to the corresponding authors.

## Supplementary material

Supplementary Figure S1 Covariate evaluations based on the final basic model. **(A)** η_CL_ versus the corresponding covariate. **(B)** η_V1_ versus the corresponding covariate. **(C)** η_Q_ versus the corresponding covariate. **(D)** η_V2_ versus the corresponding covariate. η represents inter-individual random effect. The solid blue lines represent the trend lines.
