# Supplementary figures and images for: Population pharmacokinetic analysis and dosing regimen optimization of teicoplanin in critically ill patients with sepsis
Source: Front Pharmacol. 2023 Apr 28;14:1132367. doi: 10.3389/fphar.2023.1132367 (PMC10175687; doi:10.3389/fphar.2023.1132367)

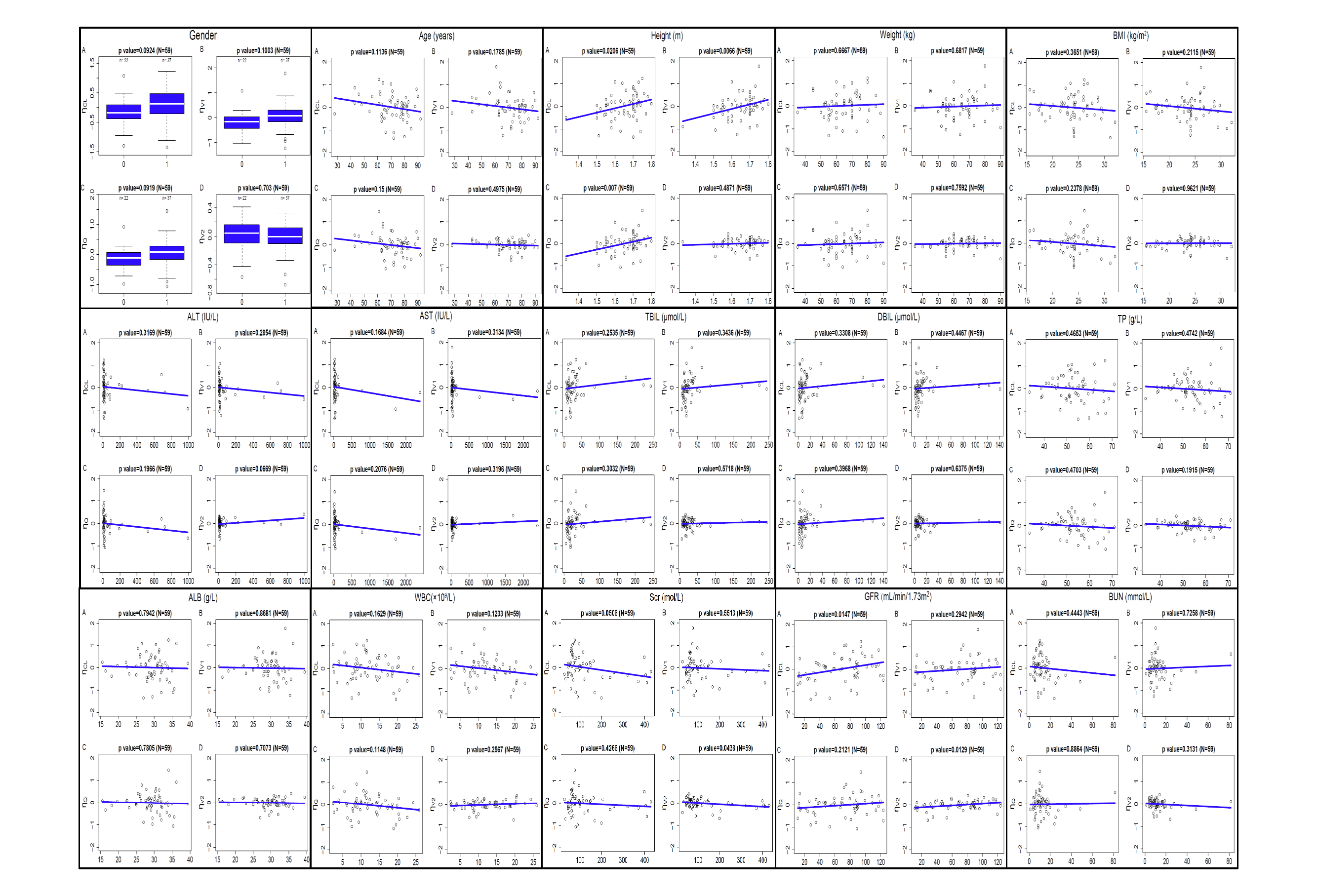

Supplement: Supplementary file 1 [file Image1.tiff]
