# Supplementary material for: Population pharmacokinetic analysis and dosing regimen optimization of teicoplanin in critically ill patients with sepsis
Source: Front Pharmacol. 2023 Apr 28;14:1132367. doi: 10.3389/fphar.2023.1132367 (PMC10175687; doi:10.3389/fphar.2023.1132367)
Supplement: Supplementary file 2 [file Table1.docx]

**Supplementary data:**

**Table S1. Impact of covariates on the Bayesian posthoc PK parameters from the final PPK basic model**

|  | **η_CL_** | **η_V1_** | **η_Q_** | **η_V2_** | **N** |
| --- | --- | --- | --- | --- | --- |
| **BUN** | 0.4443 | 0.7258 | 0.8864 | 0.3131 | 59 |
| **GFR** | **0.0147** | 0.2942 | 0.2121 | **0.0129** | 59 |
| **Scr** | 0.0506 | 0.5513 | 0.4266 | **0.0438** | 59 |
| **WBC** | 0.1629 | 0.1233 | 0.1148 | 0.2567 | 59 |
| **ALB** | 0.7942 | 0.8681 | 0.7805 | 0.7073 | 59 |
| **TP** | 0.4653 | 0.4742 | 0.4703 | 0.1915 | 59 |
| **DBIL** | 0.3308 | 0.4467 | 0.3968 | 0.6375 | 59 |
| **TBIL** | 0.2535 | 0.3436 | 0.3032 | 0.5718 | 59 |
| **AST** | 0.1684 | 0.3134 | 0.2076 | 0.3196 | 59 |
| **ALT** | 0.3169 | 0.2854 | 0.1966 | 0.0669 | 59 |
| **BMI** | 0.3651 | 0.2115 | 0.2378 | 0.9621 | 59 |
| **Weight** | 0.6667 | 0.6817 | 0.6571 | 0.7592 | 59 |
| **Height** | **0.0206** | **0.0066** | **0.0070** | 0.4871 | 59 |
| **Age** | 0.1136 | 0.1785 | 0.1500 | 0.4975 | 59 |
| **Sex** | 0.0924 | 0.1003 | 0.0919 | 0.7030 | 59 |

η_CL,_ η_V1_, η_Q_, η_V2_ represent the individual random effect of CL, V1, Q and V2, respectively, N is the sample size, values in the table are P values, and bold fonts represent covariates with significant effects (P < 0.05).
